# Immune cells mediate the effect of plasma lipidomes on IgA nephropathy: a Mendelian randomization study

**DOI:** 10.1080/0886022X.2025.2498631

**Published:** 2025-05-06

**Authors:** Quanxin Li, Ye Chen, Yahan Zhu, Xiaoyang Cui, Jichen Pan, Xiao Li, Xiaolin Liu

**Affiliations:** ^a^ Department of Clinical Laboratory, The Second Hospital, Cheeloo College of Medicine, Shandong University, Jinan, Shandong, China; ^b^ Department of Nephrology, The First Affiliated Hospital of Zhengzhou University, Zhengzhou, China; ^c^ School of Medicine, Zhengzhou University, Zhengzhou, Henan, China; ^d^ Department of Pharmacy, The Second Hospital, Cheeloo College of Medicine, Shandong University, Jinan, Shandong, China; ^e^ National Key Laboratory for Innovation and Transformation of Luobing Theory, The Key Laboratory of Cardiovascular Remodeling and Function Research, Chinese Ministry of Education, Chinese National Health Commission and Chinese Academy of Medical Sciences, Department of Cardiology, Qilu Hospital of Shandong University, Jinan, China

**Keywords:** IgA nephropathy, genome-wide association study, Mendelian randomization, causal relationship, lipid metabolism, immune modulation

## Abstract

**Background:**

IgA nephropathy (IgAN) is a leading cause of chronic kidney disease, often associated with dyslipidemia and immune dysfunction. This study employs Mendelian randomization (MR) to investigate the causal relationship between plasma lipidomes and IgAN, with a focus on the potential mediating role of immune cells.

**Methods:**

We analyzed the 179 genetically predicted plasma lipidomes and the IgAN gene using two-sample Mendelian randomization (TSMR) and multivariable MR based on summary-level data from a genome-wide association study, and the results were validated by liquid chromatography-mass spectrometry. Furthermore, we quantified the proportional effect of immune cell-mediated lipidomes on IgAN using TSMR.

**Results:**

This study identified significant causal relationships of 3 lipidomes on IgAN risk by examining 179 lipidome traits as exposures. To investigate whether the impact of the 3 lipid groups on IgAN is specific, we performed TSMR analyses using 3 lipidomes as exposure factors and 4 nephritides as outcomes. Specifically, only phosphatidylinositol (18:1_20:4) was found to have a significant negative relationship with IgAN incidence (IVW method, *p* = 0.01, OR = 0.71, 95% CI = 0.55 - 0.92). Our further analysis focused on 8 immune cells associated with IgAN. We identified 2 immune cell phenotypes that may contribute to phosphatidylinositol (18:1_20:4)-mediated IgAN by careful screening.

**Conclusions:**

Our findings provide robust genetic evidence supporting a causal link between plasma lipidomes and IgAN, with immune cells acting as potential mediators. Phosphatidylinositol (18:1_20:4) emerges as a promising biomarker for IgAN risk stratification, early detection, and therapeutic intervention. Modulating its plasma levels may offer novel avenues for IgAN management.

## Introduction

IgAN represents the most prevalent form of chronic kidney disease (CKD) and constitutes a significant etiological factor in the progression to end-stage renal disease (ESRD) [1,2]. Currently, renal puncture biopsy is the most dependable technique for diagnosing IgAN [3]. However, not all patients approve of this intrusive test. There is a lack of noninvasive diagnostic indicators that specifically target IgAN. Common IgAN treatment drugs include endothelin receptor antagonists, renin-angiotensin-aldosterone receptor inhibitors, sodium-glucose transporter 2 inhibitors, and glucocorticoids [4–7]. However, poor sensitivity and adverse effects remain great challenges in the treatment of IgAN. Therefore, the search for new diagnoses and therapeutic targets for IgAN has become the focus of nephrology research.

CKD is frequently associated with dyslipidemia and particular liposomes play an important role in the diagnosis and therapy of CKD [8]. The dyslipidemic profile associated with CKD is characterized by elevated triglyceride concentrations, reduced high-density lipoprotein (HDL) cholesterol levels, and either normal or marginally decreased low-density lipoprotein (LDL) cholesterol levels [9]. Lipidomics has revealed the association of triacylglycerols species containing linolenic acid with body mass index and prognosis in patients with IgAN [10]. However, there are currently no distinct serologic markers to distinguish IgAN from other nephritis, such as chronic and acute tubulo-interstitial nephritis, glomerulonephritis, and Henoch-Schönlein purpura nephritis (HSPN). The identification of particular circulating liposome markers can contribute to the identification and management of IgAN. The recognized ‘four-hit’ theory of IgAN pathogenesis suggests that immune cells play a significant role in IgAN development, and whether circulating liposomes regulate IgAN by influencing immune cell function remains a mystery [11].

Notably, gene prediction using genome-wide association study (GWAS) data seems to have promise for assisting us in resolving the issue. MR is a research strategy that analyzes genetic variants associated with exposure to determine the causal relationship between exposure and illness outcome [12,13]. Single-nucleotide polymorphisms (SNPs) are used as instrumental variables in MR to examine the causal effect of exposures on outcomes. The utilization of MR is gaining traction as a method for investigating potential causal relationships between exposure factors and diseases because it stimulates the randomization process of causal reasoning in randomized controlled trials, making the design less vulnerable to confounding and reversing causal bias.

Therefore, this study aimed to investigate the correlation between circulating liposomes and IgAN by MR to identify specific liposomes associated with IgAN and evaluate the extent to which immune cells mediate the effects of circulating liposomes on IgAN. In this study, we aim to identify plasma biomarkers for the noninvasive diagnosis and therapy of IgAN.

## Methods

### Study design

**Figure 1.**
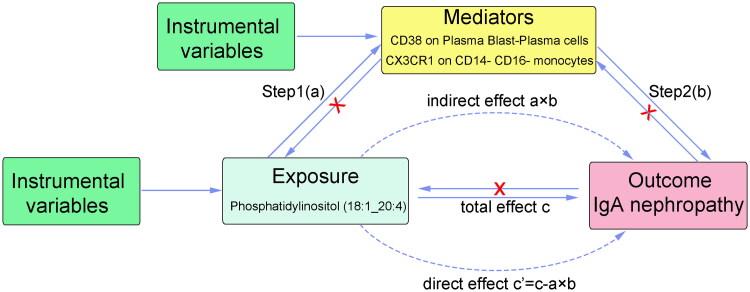
Diagrams illustrating associations examined in this study. (A) The total effect between 179 lipidomes and IgAN. c is the total effect using genetically predicted 179 lipidomes as exposure and IgAN as outcome. (B) The total effect was decomposed into: (i) indirect effect using a two-step approach (where a is the total effect of 179 lipidomes on 731 immune cells, and b is the effect of 731 immune cells on IgAN) and the product method (a × b) and (ii) direct effect (c′ = c – a × b). Proportion mediated was the indirect effect divided by the total effect. (C) Red X indicates that there is no reverse causation.

The MR analysis of this study was predicated on immune cells regulating circulating liposome-mediated IgAN. This analysis used comprehensive GWAS summary datasets and gained participant agreement in the original trials, eliminating the need for further ethical approval. The simplified framework of MR for our study is illustrated in Figure 1.

### Description of the data sources

In this study, all data used in the GWAS was publically available, and all of the participants were Europeans. Lipidome data were derived from Linda Ottensmann’s GWAS catalog, which included 179 lipid species and 7174 Finnish individuals [14,15]. The data for IgAN, chronic and acute tubulointerstitial nephritis, glomerulonephritis, and HSPN were extracted from the FennGenn consortium GWAS summary data sources. To ensure the accuracy of the results, we also validated them using the IgAN dataset (GCST90018866) from the Integrative Epidemiology Unit (IEU) OpenGWAS Databases [16]. Furthermore, the genetic data for 731 immune cells were obtained from the GWAS Public Catalog [17,18]. All GWAS data utilized in this study were sourced from various consortia and organizations, and we used MRlap to analyze sample overlap and its effect as a modifier of these biases, ensuring no sample overlap. Since the data were obtained from the abstract level of GWAS studies, ethical approval and informed consent were secured by the original researchers. Detailed information regarding these approvals is provided in Supplementary Table S1.

### Selection of genetic instrumental variables

Selection criteria for plasma lipidome and immune cell IVs were independent single-nucleotide polymorphisms (SNPs) with genome-wide significance (r^2^ = 0.001, KB = 10000, *p* < 1 × 10^−6^) [19]. When r^2^ > 0.001 and kb < 10000, SNPs are excluded from the current analysis to ensure the absence of linkage disequilibrium (LD) correlation [20]. In addition, we quantified the strength of the genetic instrument for all SNPs with an F-statistic calculated as (β^2^/se^2^) and as a follow-up analysis for instrumental variables (IVs) with an F-statistic higher than 10. The heterogeneity test excluded significantly heterogeneous SNPs with an F statistic < 10, retaining effective SNPs as instrumental variables (Supplementary Tables S9–15) [21].

### MR analysis

To carry out the MR analysis, we utilized R software (version 4.3.2, http://www.r-project.org), the ‘MendelianRandomization’ package, the ‘meta’ package, and the ‘Two-Sample MR’ package (version 0.5.8) [22]. To predict the impact of plasma lipidomes on IgAN, we used three complementary methods for the univariate MR analysis: IVW, Weighted Median, and MR-Egger. For further verification, the results were subjected to Bayesian weighted MR (BWMR). IVW was widely regarded as the principal approach for estimating causal relationships because it provides accurate results if all selected SNPs are valid IVs. We further performed a meta-analysis of IVW results from FinnGen and IEU datasets and employed multivariable MR to help address potential pleiotropy and confounding effects. Considering the effect of sample overlap, we used MRlap (https://github.com/n-mounier/MRlap). MRlap is a relatively new method, that considers a number of biases that MR analyses can be subject to [23]. MR estimates are subject to winner’s curse, which occurs when the same sample is used to select IVs and estimate their effect on the exposure. MRlap corrects for weak instrument bias and winner’s curse, whilst accounting for sample overlap and its effect as a modifier of these biases. MRlap computes a test statistic to determine whether the corrected effect estimate significantly deviates from the IVW observed effect. In the absence of a significant difference, the IVW-MR estimate can be reliably utilized [24,25].

### Reverse MR analysis

Swapping exposures and endings, we performed a two-sample univariate MR analysis to test the directionality of the causal effects and confirm our judgment regarding causal directionality according to the results of the analysis [18].

### Mediated MR analysis

Using the data for IgAN from the FennGenn datasets as outcome data sources, we employed a two-step MR approach to investigate the potential mediating role of immune cells in the causal pathway linking lipidomes to IgAN (Figure 1). The total effect was partitioned into two distinct components: the indirect effect, which operates through mediators, and the direct effect, which occurs independently of mediators [26]. The overall impact of lipidomes on IgAN was analyzed by separating it into two components: the direct effects of lipidomics on IgAN (c’ in Figure 1) and the indirect effects mediated by lipidomics through a mediator (a x b in Figure 1). Using the indirect effect divided by the total effect, we determined how much of the direct effect is mediated by the mediating effect. In addition, delta confidence intervals were calculated using 95% confidence intervals [22].

### Sensitivity analysis

It is imperative to conduct the sensitivity analysis in order to assess heterogeneity and potential pleiotropy that may substantially compromise the requirements of MR analysis. Horizontal pleiotropy can arise when instrumental variables exert effects on outcomes through mechanisms unrelated to the exposure of interest. This study used various techniques to validate the results, including the Cochran Q test, the MR-Egger intercept test, and the MR-Pleiotropy RESidual Sum and Outliers (MR-PRESSO) method [18]. In cases of heterogeneity, a Cochran Q test with *p* < 0.05 is considered significant [27]. MR-Egger intercept was used to determine the invalidity offset caused by the IV [28]. In addition, we used the MR-PRESSO method to reexamine the study for potential horizontal pleiotropy [29].

### Blood sample collection, preparation and metabolomics analyses

Plasma samples were obtained from a healthy population and from patients with pathologically confirmed IGA nephropathy. The samples were obtained from the Department of Clinical Laboratory, the Second Hospital, Cheeloo College of Medicine, Shandong University (Supplementary Table S16).

Baseline plasma collection was performed with morning blood samples (3 mL) drawn from fasting subjects’ antecubital veins into EDTA-lined polypropylene tubes. Following a 10-min centrifugation at 3000 rpm and 4 °C, plasma was extracted and stored at −80 °C until detection by liquid chromatography-mass spectrometry (LC-MS). The preparation of samples, along with LC-MS detection and the subsequent data processing and validation, adhered to established protocols as delineated in previous literature [30]. Metabolite identification was conducted by comparing accurate mass and tandem mass spectrometry results against databases including the Human Metabolome Database (HMDB, http://www.hmdb.ca), MassBank (http://www.massbank.jp/), the Kyoto Encyclopedia of Genes and Genomes (KEGG, https://www.genome.jp/kegg/), LipidMaps (http://www.lipidmaps.org), mzCloud (https://www. mzcloud.org), and a proprietary database from Panomix Biomedical Tech Co., Ltd. (Suzhou, China).

## Results

### Causal relationship between 179 lipidomes and IgAN

**Figure 2.**
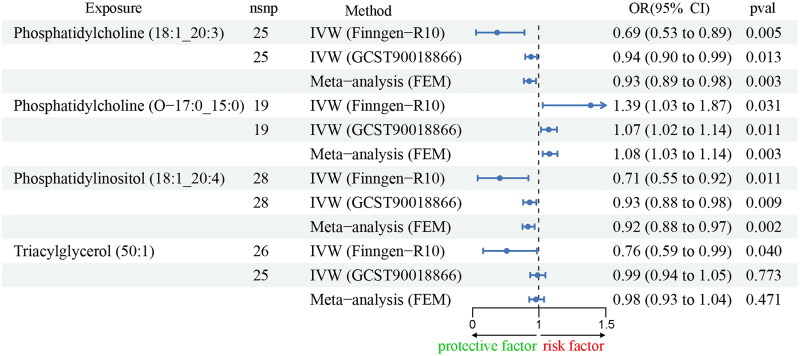
Forest Plot of Mendelian randomization analyses of 179 lipidomes on IgA nephropathy. OR, odds ratio. 95% CI, 95% confidence interval. nsnp, number of single nucleotide polymorphisms. IVW, inverse variance weighting. FEM, fixed effect model.

**Figure 3.**
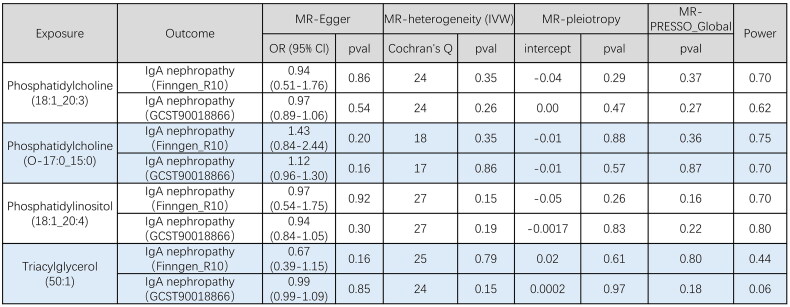
Results of multiplicity and sensitivity analyses of 4 liposomes. IVW, inverse variance weighting.

**Figure 4.**
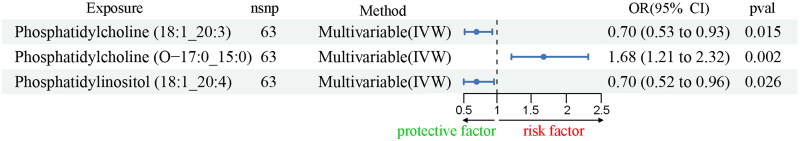
Multivariable MR of 3 liposomes and risk of IgAN. OR, odds ratio. 95% CI, 95% confidence interval. nsnp, number of single nucleotide polymorphisms. IVW, inverse variance weighting.

Using the data for IgAN from the FennGenn datasets as outcome data sources, we evaluated the causal effects of lipidomes on IgAN using TSMR. The results indicated that 4 lipidomes could potentially impact IgAN risk, including phosphatidylcholine, phosphatidylinositol, and triacylglycerol. We further validated this result using IgAN data from the IEU datasets and performed a meta-analysis of IVW estimates from FinnGen and IEU datasets. Specifically, phosphatidylinositol was identified as a protective factor, while various forms of phosphatidylcholine exhibited varying effects on IgAN (Figures 2 and 3, Supplementary Figures S1–S4). The IVW results serve as our main reference metric. Consistency in the direction of causal relationships determined by the other two analysis methods, along with a significance level of *p* < 0.05, would significantly bolster our confidence in establishing causal relationships. Both TSMR and BWMR analyses yielded entirely consistent results (Supplementary Table S2). To account for potential confounding factors among the 3 lipidomes, we conducted a multivariable MR analysis, the results revealed that 3 lipidomes maintained an independent role (Figure 4, Supplementary Table S2). To validate the directionality of causal effects, we conducted two-sample univariate MR analyses using IgAN as an exposure factor and 3 plasma lipidomes as outcomes. IgAN was also found not to affect them (Supplementary Table S3).

To investigate whether the impact of the 3 lipid groups on IgAN is specific, we performed TSMR analyses using 3 lipidomes as exposure factors and 4 nephritides as outcomes, including chronic tubulointerstitial nephritis, acute tubulointerstitial nephritis, glomerulonephritis, and Henoch-Schönlein purpura nephritis (HSPN). We found that Phosphatidylinositol (18:1_20:4) has no causal effects on them, suggesting the specificity of its association with IgAN (Supplementary Table S4).

### Causal relationship between 731 immune cells and IgAN

**Figure 5.**
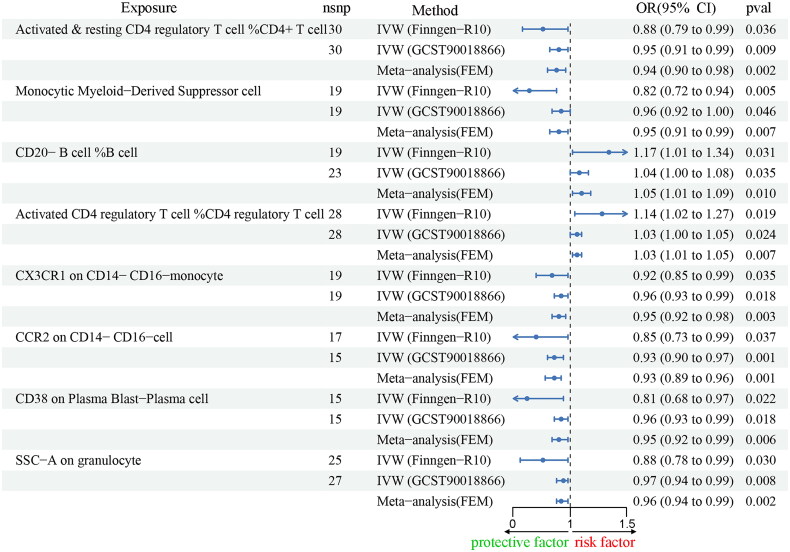
Forest Plot of Mendelian randomization analyses 731 immune cells on IgA nephropathy. OR, odds ratio. 95% CI, 95% confidence interval. nsnp, number of single nucleotide polymorphisms. IVW, inverse variance weighting. FEM, fixed effect model.

To investigate the potential impact of immune cell phenotype on the development of IgAN, we conducted a detailed statistical analysis. Using the data for IgAN from the FennGenn datasets as outcome data sources, we found that 45 immune cell phenotypes were significantly associated with IgAN, as demonstrated by *p* < 0.05 (IVW). Subsequently, we eliminated exposure factors with inconsistent OR values using the Weighted Median and MR Egger methods. By conducting sensitivity analyses, we identified 6 immune cells exhibiting significant heterogeneity or pleiotropy. To improve the robustness of our findings, we applied FDR correction to the IVW results, allowing us to detect exposure factors with an FDR-adjusted *p* > 0.05. We conducted additional validation of these findings utilizing IgAN data from the IEU datasets and subsequently performed a meta-analysis of the IVW estimates derived from both the FinnGen and IEU datasets. Consequently, we identified 8 immunophenotypes causally associated with IgAN (Figure 5, Supplementary Table S5).

### Mediating role of immune cells in phosphatidylinositol (18:1_20:4) to IgAN

**Figure 6.**
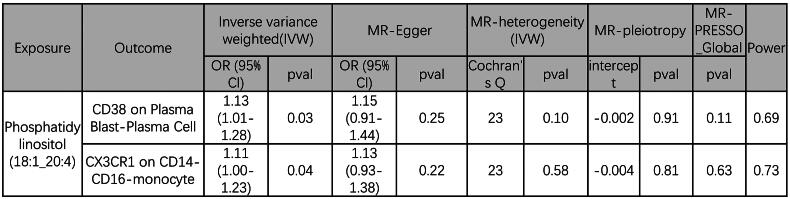
MR analysis of phosphatidylinositol (18:1_20:4) on 2 immune cells. IVW, inverse variance weighting.

**Figure 7.**
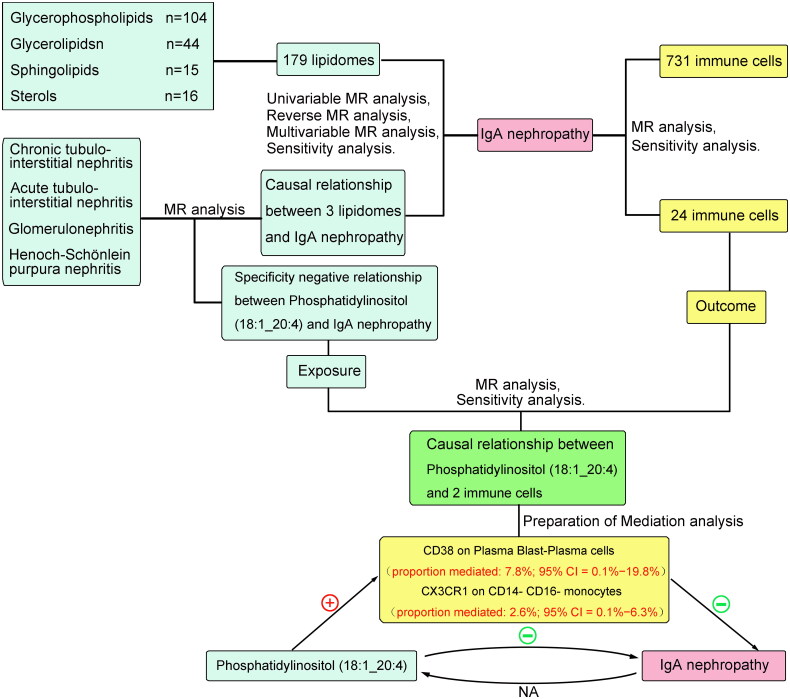
The mediation effect of immune cells in the causal effect of phosphatidylinositol (18:1_20:4) on IgAN risk.

The causal effects of Phosphatidylinositol (18:1_20:4) associated with 8 immune cells related to IgAN were evaluated through TSMR to determine which immune cells may be potential mediators. In summary, we have ultimately discovered that CD38 on Plasma Blast-Plasma cells and CX3CR1 on CD14- CD16- monocytes can act as mediators in the pathway from phosphatidylinositol (18:1_20:4) to IgAN (Supplementary Table S6, Supplementary Figure S5–S8). We found that phosphatidylinositol (18:1_20:4) was associated with increased CD38 on Plasma Blast-Plasma cells (IVW method, *p* = 0.035, OR = 1.13, 95%CI = 1.01 - 1.28) and CX3CR1 on CD14- CD16- monocytes (IVW method, *p* = 0.043, OR = 1.11, 95%CI = 1.01 - 1.23), which in turn were associated with a lower risk of IgAN (CD38 on Plasma Blast-Plasma cells: IVW method, *p* = 0.022, OR = 0.81, 95%CI = 0.68 - 0.97 and CX3CR1 on CD14- CD16- monocytes: IVW method, *p* = 0.035, OR = 0.92, 95%CI = 0.85 - 0.99) (Figure 6). To ascertain the directionality of causal effects, we performed two-sample univariate MR analyses. The results demonstrated that the two immune cell types did not influence phosphatidylinositol (18:1_20:4), as detailed in Supplementary Table S7. Additionally, IgAN was found to have no impact on the two immune cell types, as shown in Supplementary Table S8. As shown in Figure 7, the result showed that CD38 on Plasma Blast-Plasma cells accounted for 7.8% of the decreased risk of IgAN associated with phosphatidylinositol (18:1_20:4) (proportion mediated: 7.8%; 95% CI = 0.1%-19.8%), and CX3CR1 on CD14- CD16- monocytes accounted for 2.6% (proportion mediated: 2.6%; 95% CI = 0.1%∼6.3%).

### Validation of the relationship between plasma lipidome and IgAN by LC-MS

**Figure 8.**
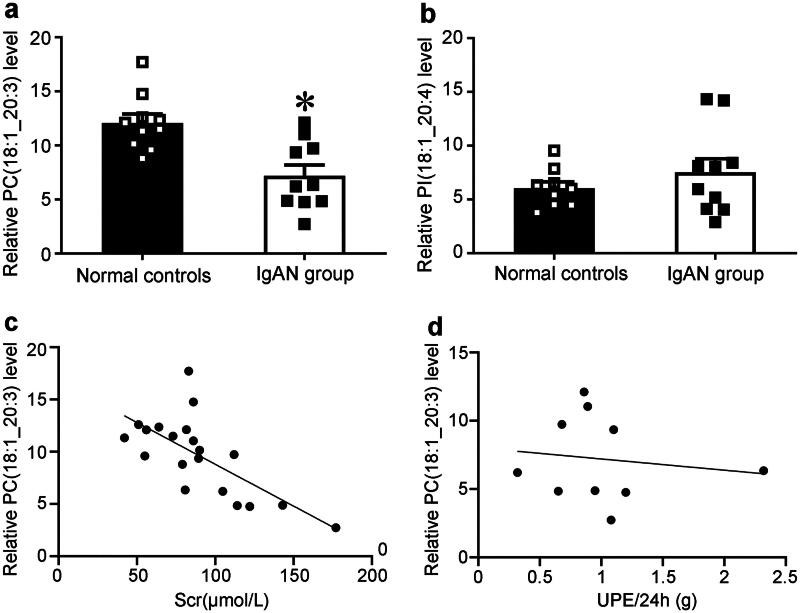
Validation of the relationship between plasma lipidome and IgAN by LC-MS. PC, Phosphatidylcholine. PI, Phosphatidylinositol. Scr, serum creatinine; UPE, urinary protein excretion.

Considering that the dataset predominantly comprises individuals of European descent, which limits the generalizability of results to other populations. In addition, independent validation experiments could increase the credibility of the results. We collected plasma samples from both healthy Chinese individuals and patients with IgAN and employed LC-MS to detect plasma liposomes. The test results indicated that, in comparison to the normal population, patients with IgAN exhibited elevated plasma levels of 107 lipids and decreased levels of 322 liposomes. Among the three liposomes previously associated with IgAN through MR, Phosphatidylcholine (18:1_20:3) demonstrated reduced levels in the plasma of IgAN patients, phosphatidylinositol (18:1_20:4) showed no significant difference, and phosphatidylcholine (O-17:0_15:0) was undetectable using LC-MS methods (Figure 8a,b, Supplementary Table S16). Subsequently, we analyzed the correlation between relative plasma liposome levels and both serum creatinine and urinary protein concentrations in IgAN patients. Our analysis revealed that phosphatidylcholine (18:1_20:3) was negatively correlated with serum creatinine (Spearman r = −0.6938, *p* < 0.01; Figure 8c) across all available subjects. Although phosphatidylcholine (18:1_20:3) also exhibited a negative correlation with urinary protein excretion, this correlation did not reach statistical significance (Figure 8d). These findings further corroborate our Mendelian randomization results, suggesting that phosphatidylcholine (18:1_20:3) may exert a protective effect against IgAN.

## Discussion

This study was the first to explore the causal relationship between the plasma lipidome and IgAN risk by a comprehensive mediation MR, which revealed that significant causal relationships of 3 lipidomes on IgAN risk and increased phosphatidylinositol (18:1_20:4) levels were associated with decreased susceptibility to IgAN, CD38 on Plasma Blast-Plasma cells and CX3CR1 on CD14- CD16- monocytes could act as mediators in the pathway from phosphatidylinositol (18:1_20:4) to IgAN. We demonstrated that the relationship between liposomes, immune cells and IgAN is unidirectional and linear by reverse MR. This analysis highlights the association between plasma lipidomes and IgAN, emphasizing the mediating role of immune cells.

We validated the results of Mendelian randomization by LC-MS methods. The results further corroborate our Mendelian randomization results, suggesting that phosphatidylcholine (18:1_20:3) may exert a protective effect against IgAN. Phosphatidylcholine is a phospholipid that is the source of choline in the body. Phosphatidylcholine is generally known to improve high blood cholesterol, slow down skin aging, and enhance brain memory. Multiple phosphatidylcholines were shown to be associated with a low risk of association with comorbid renal endpoints in a study of lipidomic analyses revealing the association of sphingolipids and phosphatidylcholines with renal impairment and all-cause mortality in patients with type 1 diabetes mellitus [31]. These results suggest that phosphatidylcholine may play a protective role in CKD. However, no correlation was observed between the other two liposomes and IgA nephropathy. This lack of association may be attributed to the assay methodology, the limited sample size, and variations in dietary habits across different ethnic groups. While the detection of liposomes *via* liquid chromatography-mass spectrometry (LC-MS) exhibits high sensitivity and specificity, certain limitations persist. Firstly, variations in lipid composition and content across different samples necessitate individualized analysis and data processing for each sample. Secondly, the high sensitivity and resolution of LC-MS make it susceptible to interference from external factors, such as sample contamination and instrument malfunction. Furthermore, the limited sample size employed to validate the results of Mendelian randomization may not adequately represent the broader Asian population. Last but not least, European and Asian populations have different dietary structures. For example, Europeans consume more beef and lamb and olive oil, while Chinese people consume more pork, soybean oil, peanut oil, and so on. These dietary differences may lead to large differences in plasma liposome levels. These findings indicate that conducting independent validation of MR outcomes within ethnically diverse populations will enhance the accuracy and generalizability of the results.

IgAN is the predominant form of primary glomerulonephritis globally, presenting a spectrum of clinical manifestations that can range from asymptomatic microscopic hematuria to acute kidney injury. The majority of patients experience a chronic progressive course, with approximately one-third progressing to CKD or even end-stage renal disease (ESRD) within a decade of disease onset. This disease significantly impacts patient survival and places a substantial burden on society. At present, the gold standard for IgAN diagnosis is still renal needle biopsy, but renal biopsy is an invasive procedure, which not all renal patients can accept due to the risk of bleeding, infection, and other adverse events; at the same time, there are also absolute/relative contraindications to renal needle biopsy in some patients, which limit its clinical application and increase the difficulty of confirming the diagnosis of IgAN. Therefore, it is imperative to find a noninvasive diagnostic method for IgAN.

Dyslipidemia is common in CKD and is found to contribute to the initiation and progression of CKD [20]. As far as we know, there has been little data to show IgAN-specific dyslipidemia patterns. In this study, we identified 3 lipids that may affect IgAN risk *via* TSMR. To find liposomes specifically associated with IgAN, we performed a TSMR analysis with 3 lipid groups as exposure factors and 4 other nephritides as outcomes. Eventually, we found that phosphatidylinositol (18:1_20:4) was specifically associated with IgAN and may play a protective role in IgAN. Phosphatidylinositol forms the membranes of cells and organelles and maintains membrane fluidity and function. It consists of an inositol head and a diacylglycerol tail, which are also the source of the important second messengers IP3 and DAG downstream of the future formation of the phosphatidylinositol pathway, and is an important molecule in the PI3K/AKT signaling pathway involved in a variety of disease processes, including cancer [32], depression [33], diabetes [34], CKD and so on [35,36]. Studies have shown that the expression of PI3K/Akt was significantly increased in patients with multiple CKD, alleviating renal inflammatory damage, apoptosis, and epithelial-mesenchymal transitions by inhibiting the PI3K/AKT signaling pathway [37–43]. The hyperactivation of the PI3K/Akt pathway was shown by Akt protein activation in peripheral blood mononuclear cells of IgAN patients [44], and pharmacological inhibition of PI3K/Akt signaling pathway significantly ameliorates renal injury and fibrosis in IgAN rats [45]. We speculate that hyperactivation of the PI3K/Akt signaling pathway of IgAN patients leads to increased cellular uptake of phosphatidylinositol from the serum, which explains that our findings revealed a negative correlation between plasma phosphatidylinositol (18:1_20:4) levels with IgAN risk. This study indicated phosphatidylinositol (18:1_20:4) may be a potential noninvasive diagnostic marker for IgAN, and modulating its levels in plasma may help treat IgAN.

Furthermore, accumulating evidence suggests that liposomes play an important role in the regulation of immune cell functions. A recent study has shown that dietary supplementation with eicosapentaenoic acid (EPA) ameliorates critical lupus symptoms, including autoantibody production and immunocomplex deposition in renal tissues. Utilizing a combination of lipidomic profiling and membrane dynamics analyses, the research elucidated that EPA modulates the lipid composition and fluidity of B-cell membranes. This alteration subsequently inhibits the differentiation of B-cells into autoantibody-producing plasma cells [46].

Because patients with IgAN still have a greater chance of relapse after renal transplantation, patients with IgA can reappear in the transplanted kidney with IgA deposition, IgA nephropathy is more inclined to be a systemic autoimmune disease in which the kidneys are the target organs [47,48]. The immune system plays a crucial role in IgAN development. The ‘four-hit’ theory is currently used to describe the pathogenesis of IgAN [49,50]. The first hit is genetic and environmental interactions in the body produce galactose-deficient pathogenic IgA1. The second hit is that Gd-IgA1 is recognized as a self-antigen in circulation and stimulates the production of the corresponding anti-Gd-IgA1 antibody. The third hit is immune recognition leads to the formation of immune complexes by combining Gd-IgA1 with its antibody. The fourth hit is that Gd-IgA1-containing complexes are deposited in the kidneys and lead to the activation of renal tissue cells, such as tethered cells, which in turn produce inflammatory factors and chemokines, among others, recruiting inflammatory cells to infiltrate into the renal tissues, activating the complement system, causing renal tissue damage and fibrosis, thus causing a series of nephropathic symptoms [51].

Another theory of B-cell mis-homing suggests that, in general, B cells are activated and migrate to the lamina propria, where they synthesize the low O-galactosylated and low-affinity polymeric mucosal-secreted IgA1, which is secreted to the mucosal surface and rarely enters the circulatory system. By contrast, bone marrow-derived plasma cells produce mostly monomeric and highly O-galactosylated IgA1. The increased levels of serum IgA1 and reduced O-galactosylation observed in individuals with IgA nephropathy may be attributed to the misexpression of homing receptors on the surface of mucosal IgA-secreting plasma cells, leading to their failure to home to the mucosal surface. Consequently, this may result in the direct release of ‘mucosal IgA’ into the bloodstream. B lymphocytes are the source of Gd-IgA1 molecules, and the study of B lymphocyte activation is therefore important for understanding the pathogenesis of IgA nephropathy. In any case, immune cells play an important role in the pathogenesis of IgAN [52–54].

In this study, we revealed that 8 immunophenotypes were causally linked to IgAN. CD38 on Plasma Blast-Plasma cells and CX3CR1 on CD14- CD16- monocytes were negatively correlated with IgAN, and those can act as mediators in the pathway from phosphatidylinositol (18:1_20:4) to IgAN. Recent studies have shown that CD38 on Plasma Blast-Plasma cells was associated with a lower risk of Parkinson’s disease [55], CX3CR1 on CD14- CD16- monocytes was associated with a lower risk of developing cerebral aneurysms [56] and was associated with a higher risk of chronic pancreatitis [57]. The two immune cells have received limited attention in the literature, and the mechanisms through which they regulate disease remain unidentified, warranting further investigation. These studies show that these immune cells play an important role in a variety of diseases. Which signaling pathways does phosphatidylinositol (18:1_20:4) utilize to control these two cellular processes? Studies have shown that inhibition of PI3K signaling *in vivo* ablates bone marrow plasma cells [58]. Hyaluronan carried by tumor-derived microvesicles induces IL-10 production in classical (CD14++CD16−) monocytes *via* PI3K/Akt/mTOR-dependent signaling pathway [59]. These studies suggest that activation of the PI3K/AKT signaling pathway promotes expression and activation of biological functions in plasma cells and monocytes. We hypothesized that phosphatidylinositol (18:1_20:4) affects the function of these two immune cells and plays a role in IgAN by regulating the PI3K/AKT signaling pathway. More biological experiments need to confirm its regulatory mechanism.

There are several noteworthy advantages to this study. Firstly, we used multiple complementary MR methods to investigate the involvement of 179 plasma lipidomes and 731 immune cell phenotypes in IgAN risk, minimizing the effects of residual confounding factors. Secondly, we searched for a plasma liposome that may be a marker for the noninvasive diagnosis of IgAN, providing a new direction for the diagnosis of IgAN. Thirdly, we explored the potential mediating impact of immune factors on the risk of IgAN, offering a significant contribution to the understanding of the mechanisms underlying risk factors associated with IgAN. Additionally, we performed independent validation on MR outcomes within ethnically diverse populations and a thorough sensitivity analysis to enhance the credibility of our findings.

Nevertheless, our study is subject to several limitations. Firstly, a less stringent threshold of *p* < 1 × 10 ^− 6^ was employed when screening lipidome SNPs, deviating from the conventional threshold of 5 × 10^–8^ to obtain a sufficient number of SNPs. Secondly, the investigation did not assess alterations in plasma liposomes in patients with IgA nephropathy before and after treatment to evaluate their potential utility as significant prognostic indicators. Thirdly, we can not state the mechanism of action of screened plasma liposomes and immune cells in IgAN, which needs to be investigated by further biological experiments. Additionally, we currently do not have access to raw data that can be used to perform nonlinear MR analysis is one of the limitations of this study.

## Conclusion

Our research suggests potential causal relationships among the plasma lipidomes, immune cells, and IgAN by using mediation MR. Specifically, CD38 on Plasma Blast-Plasma cells and CX3CR1 on CD14- CD16- monocytes mediate the regulatory effect of phosphatidylinositol (18:1_20:4) on IgAN. These results enhance our comprehension of the involvement of lipidomes and immune cells in influencing the risk of IgA nephropathy. Furthermore, our study has the potential to aid in the discovery of biochemical indicators for the prediction, screening, and early detection of IgA nephropathy, thereby paving the way for further exploration of its therapeutic target.

## Supplementary Material

Supplementary Figure S8.jpg

Figure_1_8 (1).zip

Supplementary Figure S5.jpg

Supplementary Figure S4.jpg

Supplementary Table S1 to S16.xlsx

Supplementary Figure S7.jpg

Supplementary Figure S1.jpg

Supplementary Figure S2.jpg

Supplementary Figure S3.jpg

Supplementary Figure S6.jpg

## Data Availability

The original contributions presented in the study are included in the article/Supplementary Material. All data are publicly available.
