# Supplementary figures and images for: Immune cells mediate the effect of plasma lipidomes on IgA nephropathy: a Mendelian randomization study
Source: Ren Fail. 2025 May 6;47(1):2498631. doi: 10.1080/0886022X.2025.2498631 (PMC12057791; doi:10.1080/0886022X.2025.2498631)

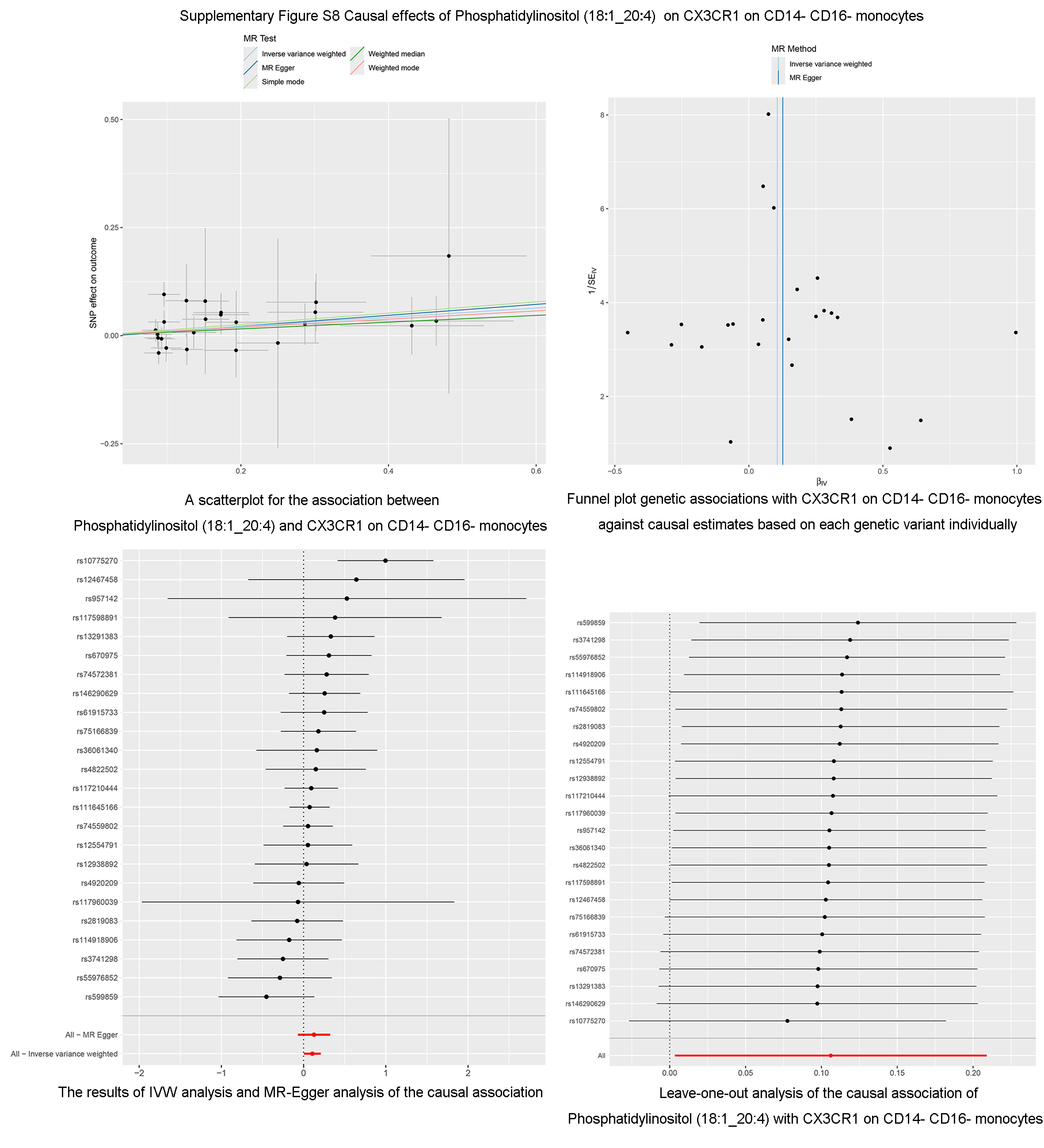

Supplement: Supplementary Figure S8.jpg [file IRNF_A_2498631_SM5576.jpg]

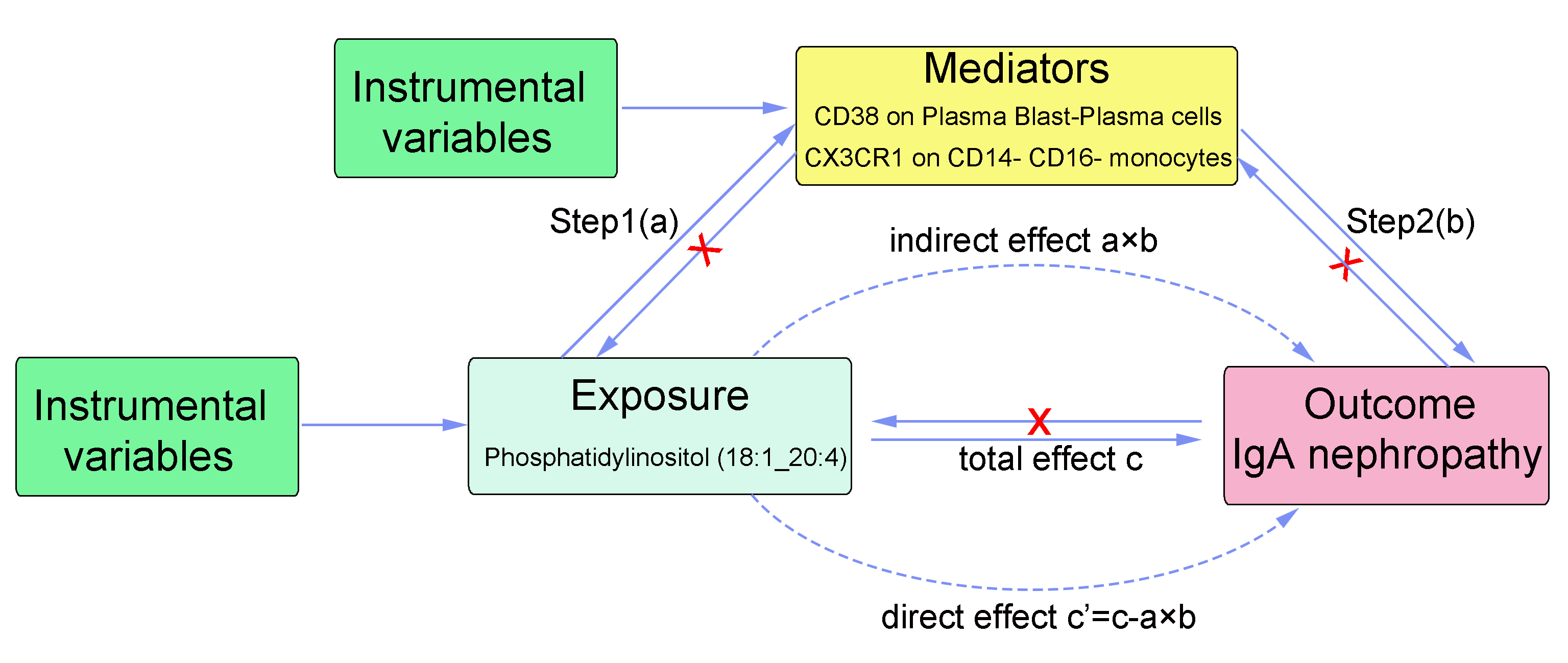

Supplement: Figure_1_8 (1).zip [file IRNF_A_2498631_SM5575.zip › Figure 1-8/Figure 1.tif]

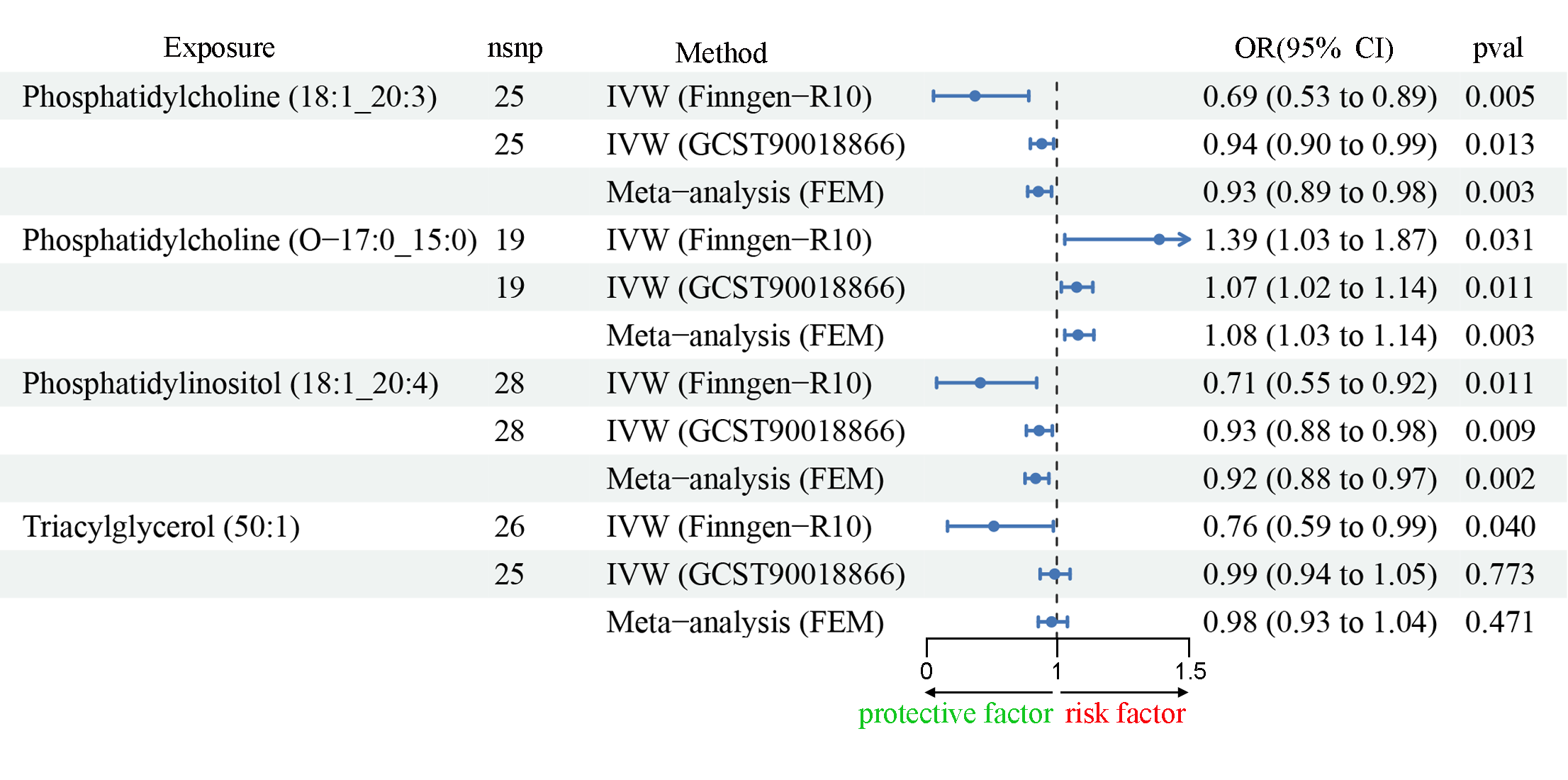

Supplement: Figure_1_8 (1).zip [file IRNF_A_2498631_SM5575.zip › Figure 1-8/Figure 2.tif]

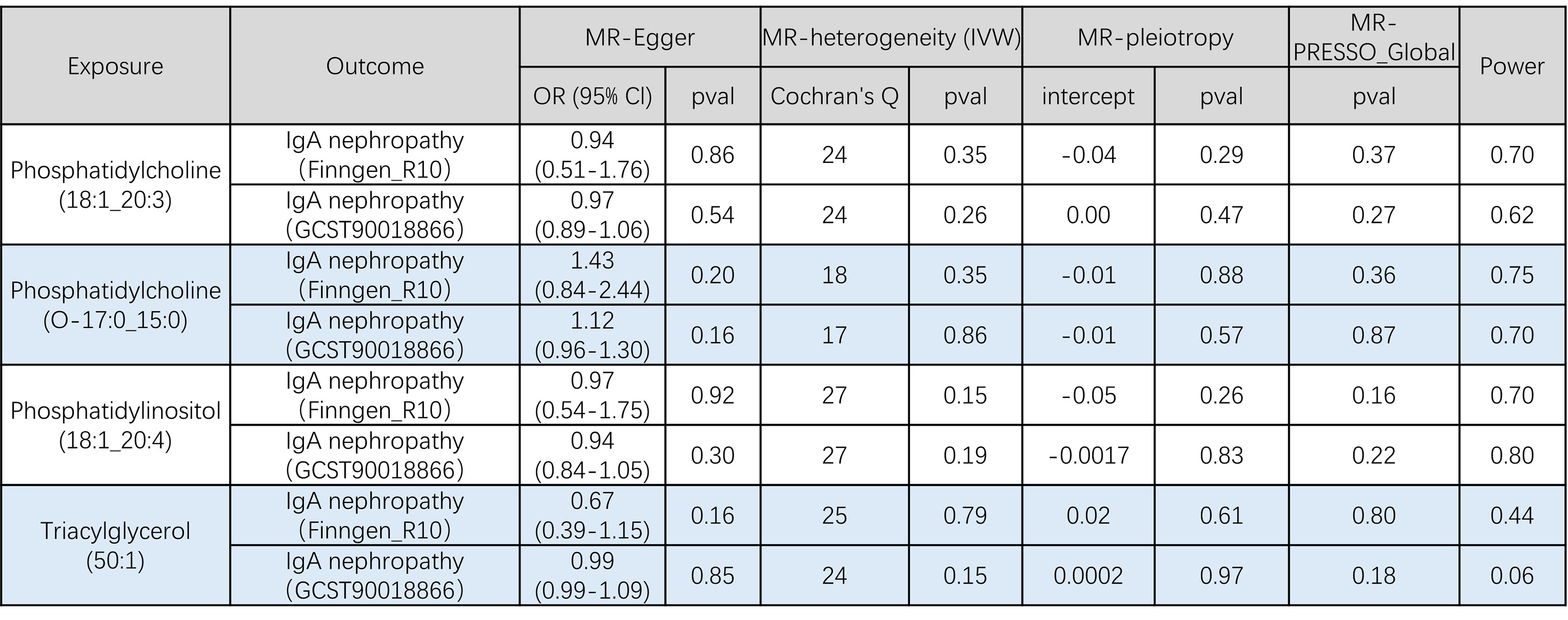

Supplement: Figure_1_8 (1).zip [file IRNF_A_2498631_SM5575.zip › Figure 1-8/Figure 3 .tif]

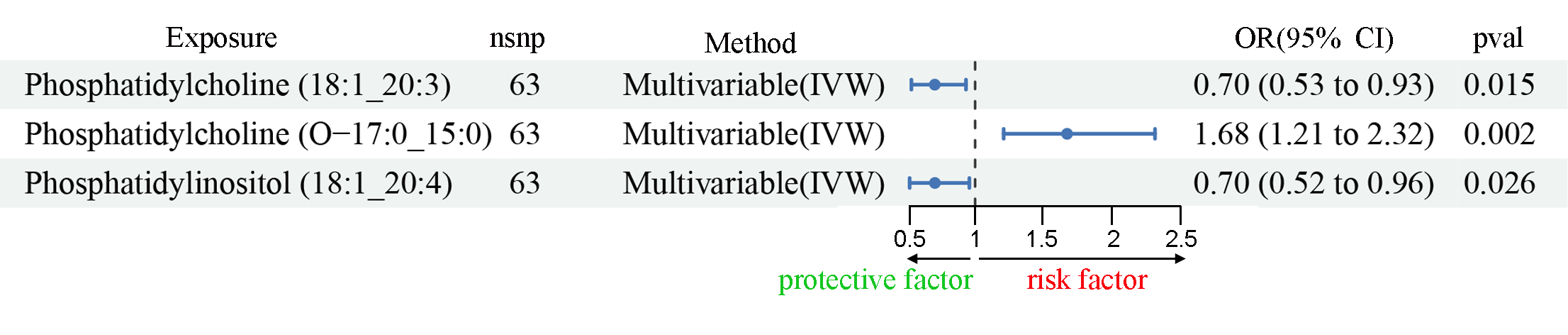

Supplement: Figure_1_8 (1).zip [file IRNF_A_2498631_SM5575.zip › Figure 1-8/Figure 4.tif]

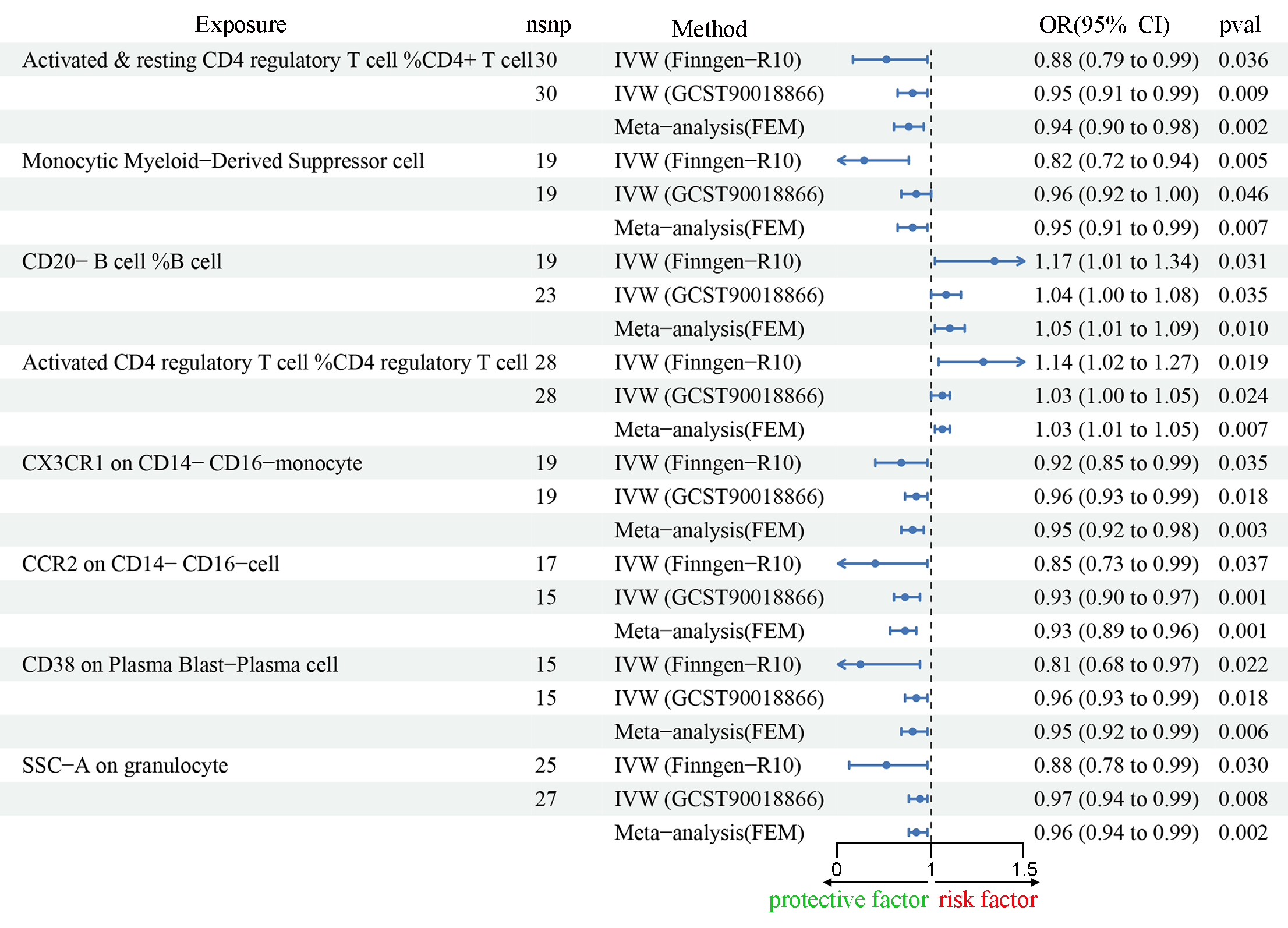

Supplement: Figure_1_8 (1).zip [file IRNF_A_2498631_SM5575.zip › Figure 1-8/Figure 5.tif]

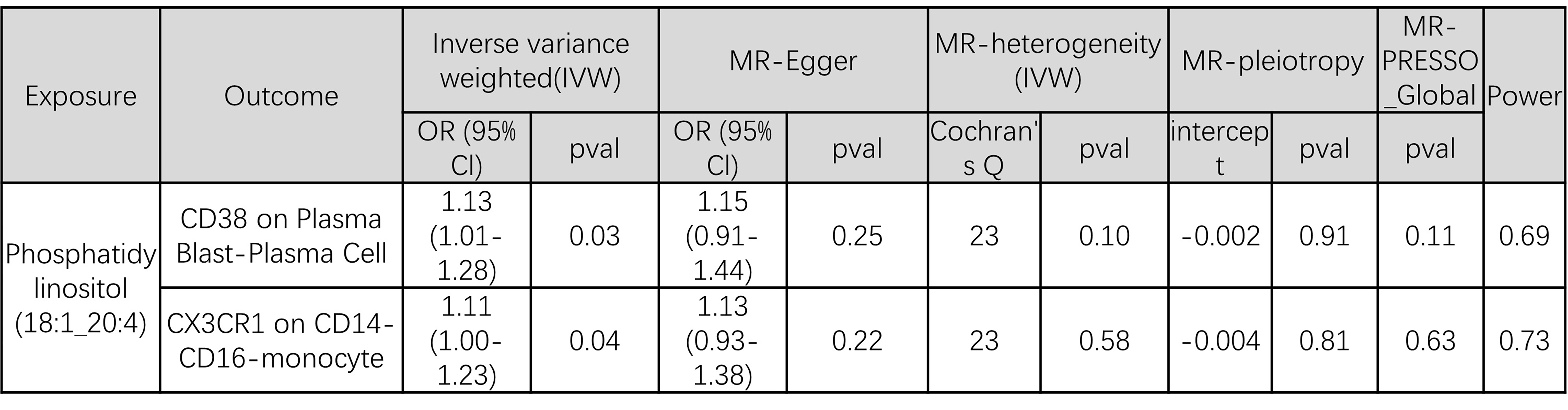

Supplement: Figure_1_8 (1).zip [file IRNF_A_2498631_SM5575.zip › Figure 1-8/Figure 6.tif]

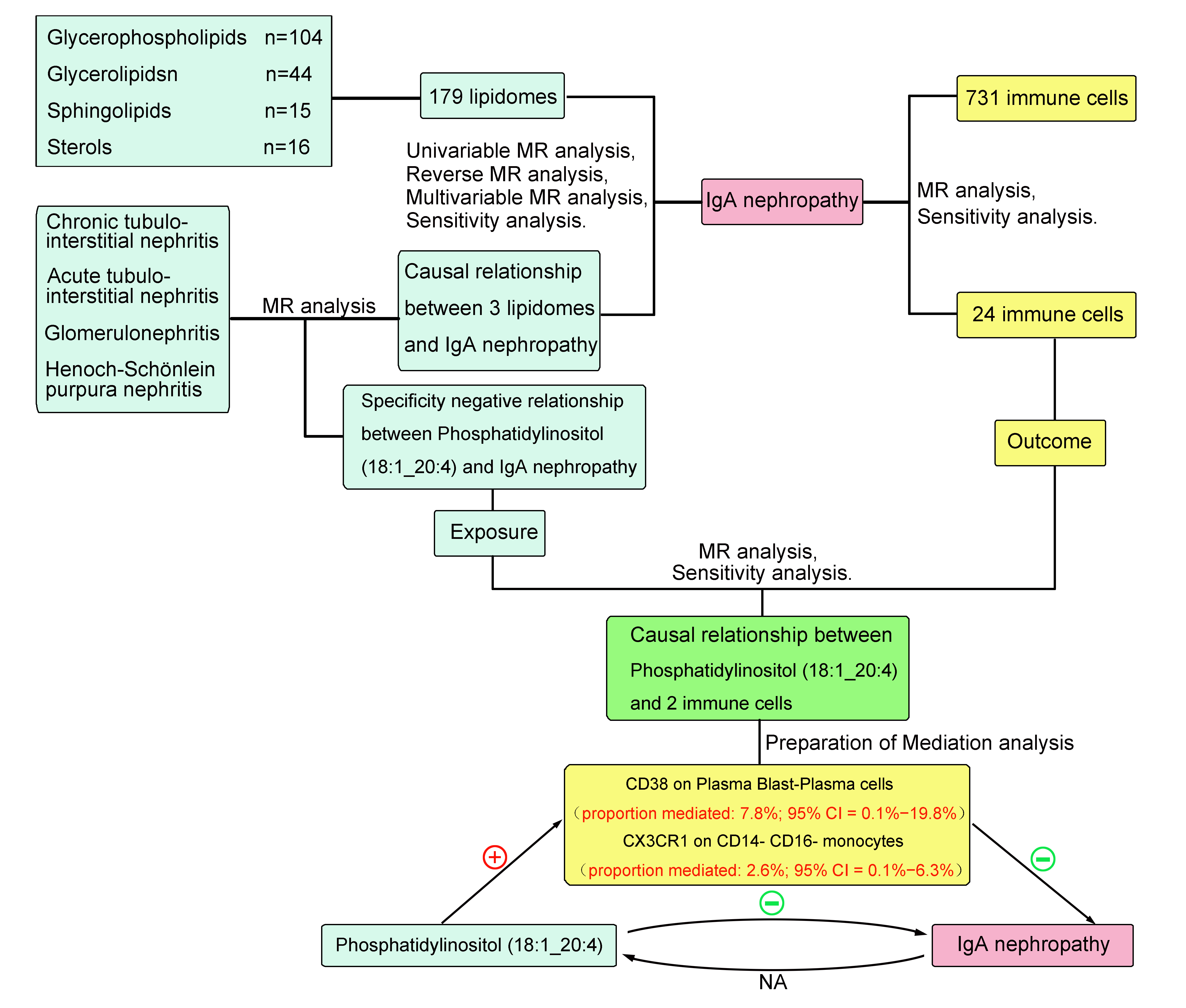

Supplement: Figure_1_8 (1).zip [file IRNF_A_2498631_SM5575.zip › Figure 1-8/Figure 7.tif]

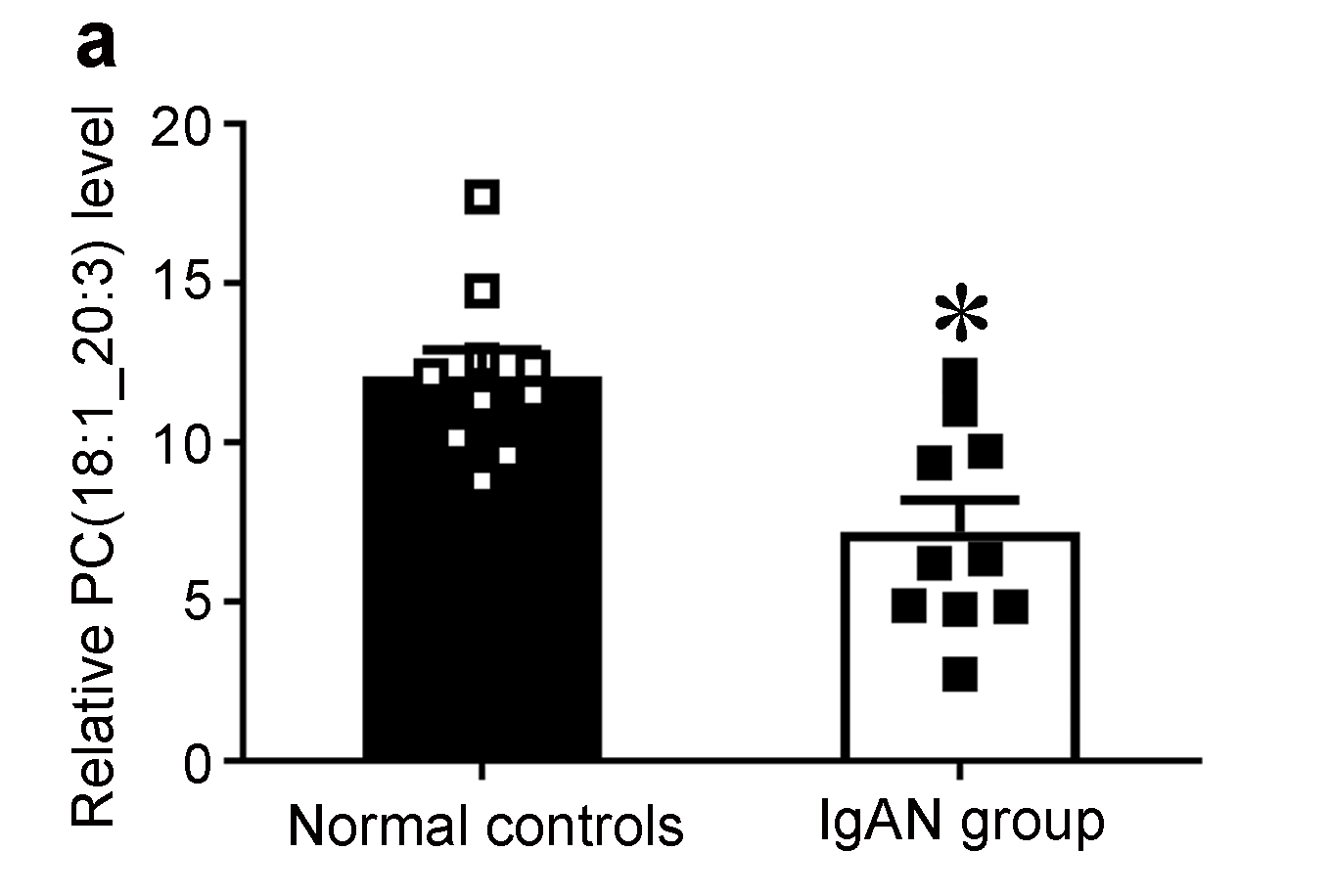

Supplement: Figure_1_8 (1).zip [file IRNF_A_2498631_SM5575.zip › Figure 1-8/Figure 8a.tif]

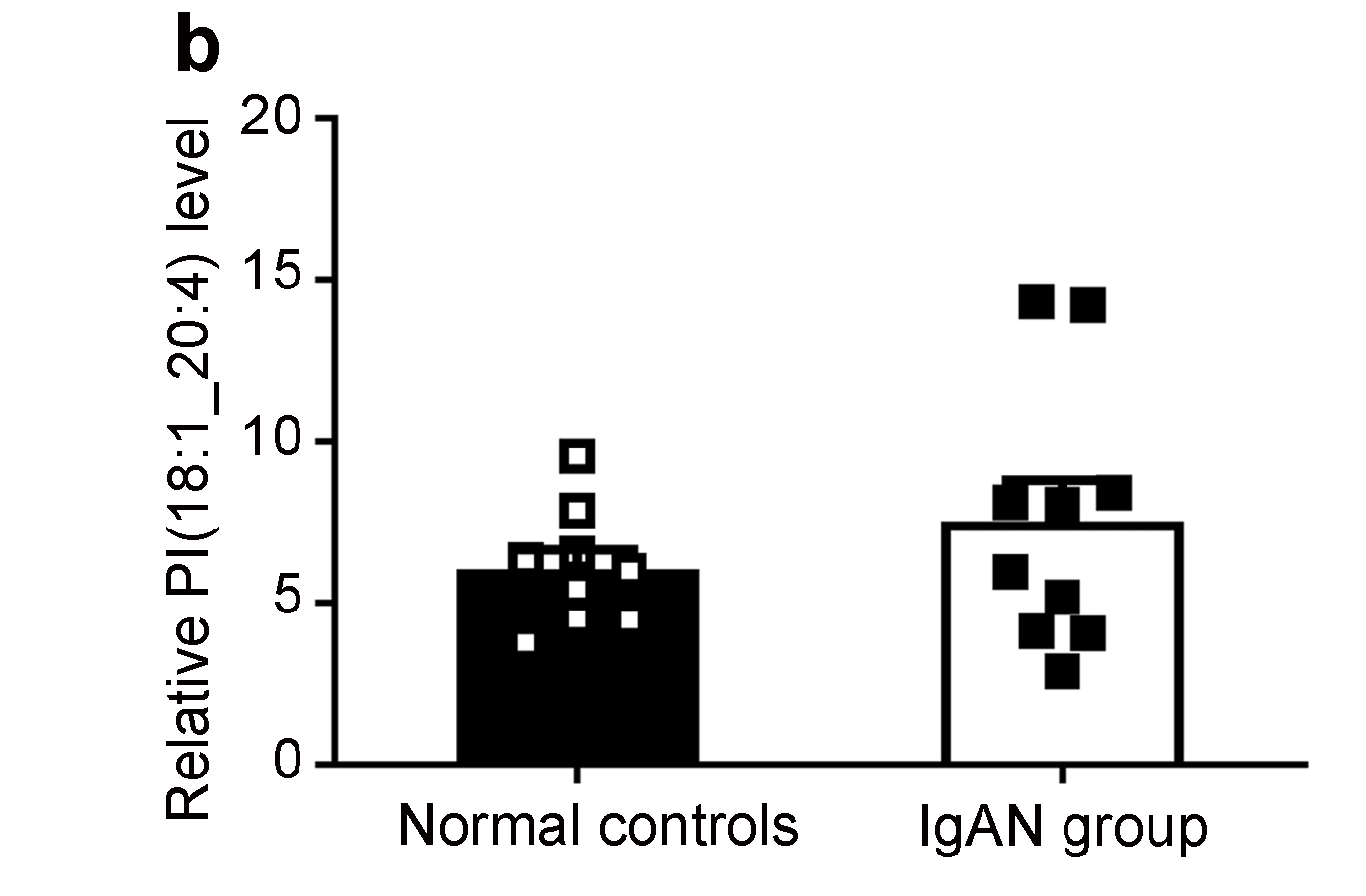

Supplement: Figure_1_8 (1).zip [file IRNF_A_2498631_SM5575.zip › Figure 1-8/Figure 8b.tif]

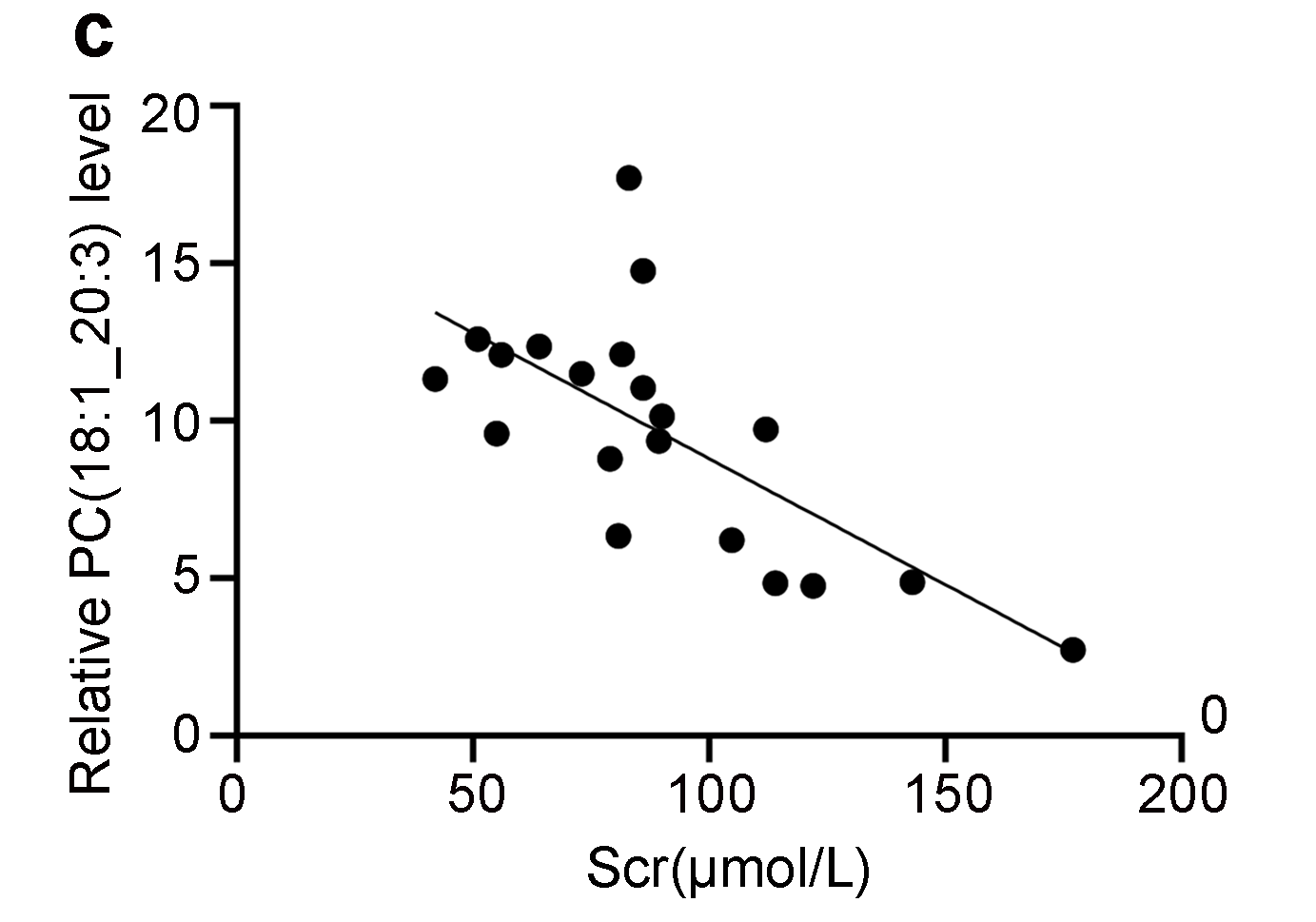

Supplement: Figure_1_8 (1).zip [file IRNF_A_2498631_SM5575.zip › Figure 1-8/Figure 8c.tif]

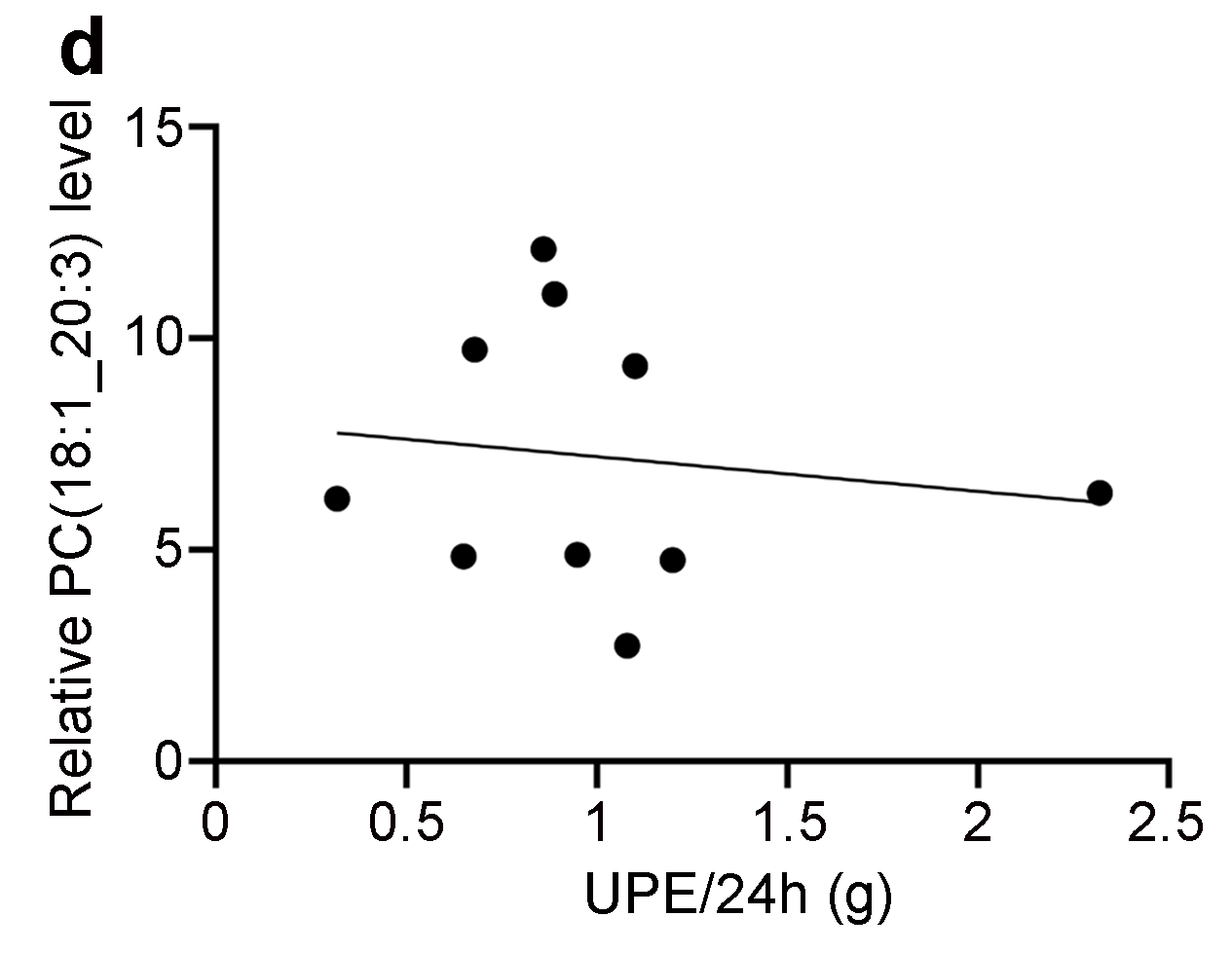

Supplement: Figure_1_8 (1).zip [file IRNF_A_2498631_SM5575.zip › Figure 1-8/Figure 8d.tif]

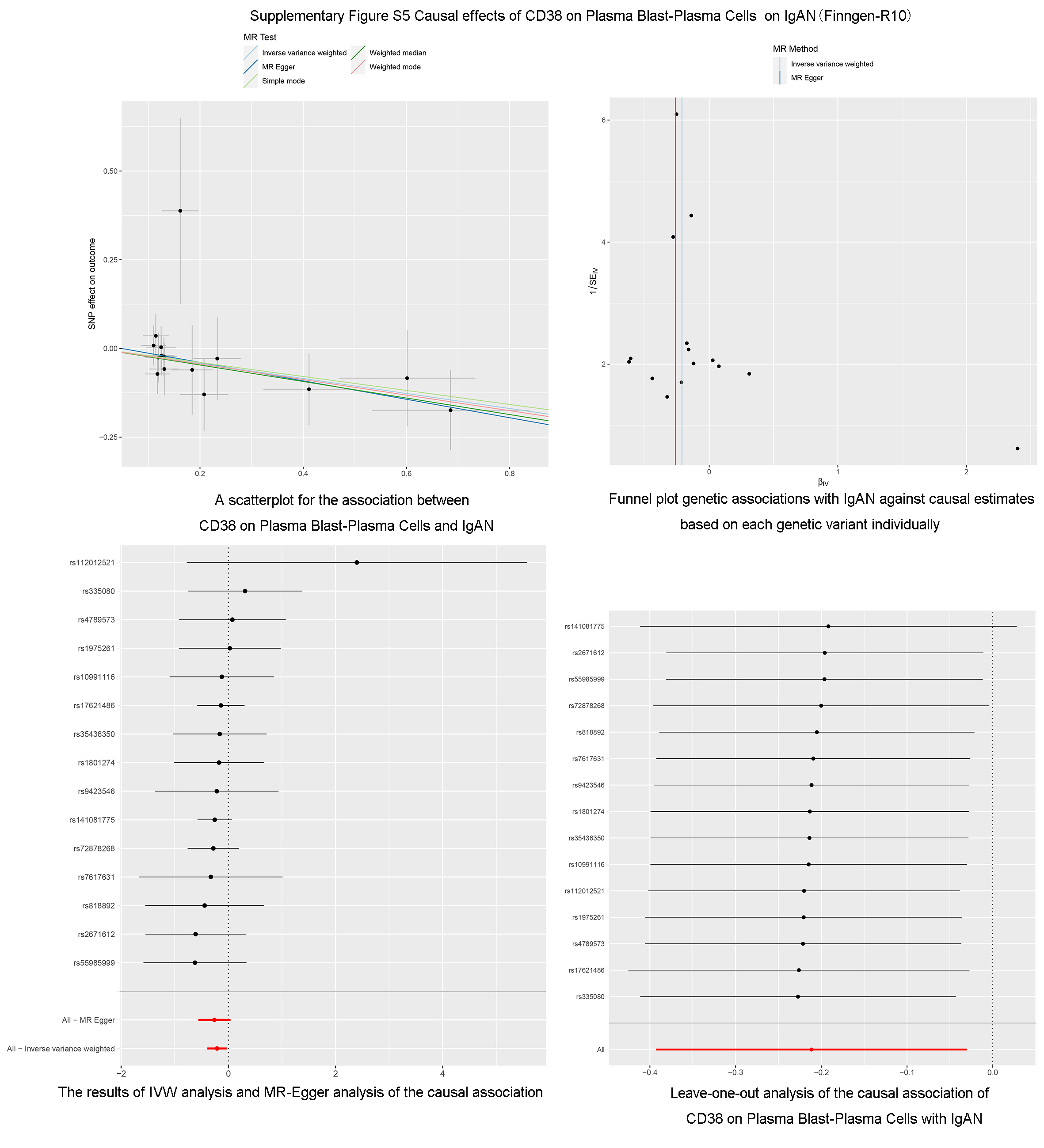

Supplement: Supplementary Figure S5.jpg [file IRNF_A_2498631_SM5574.jpg]

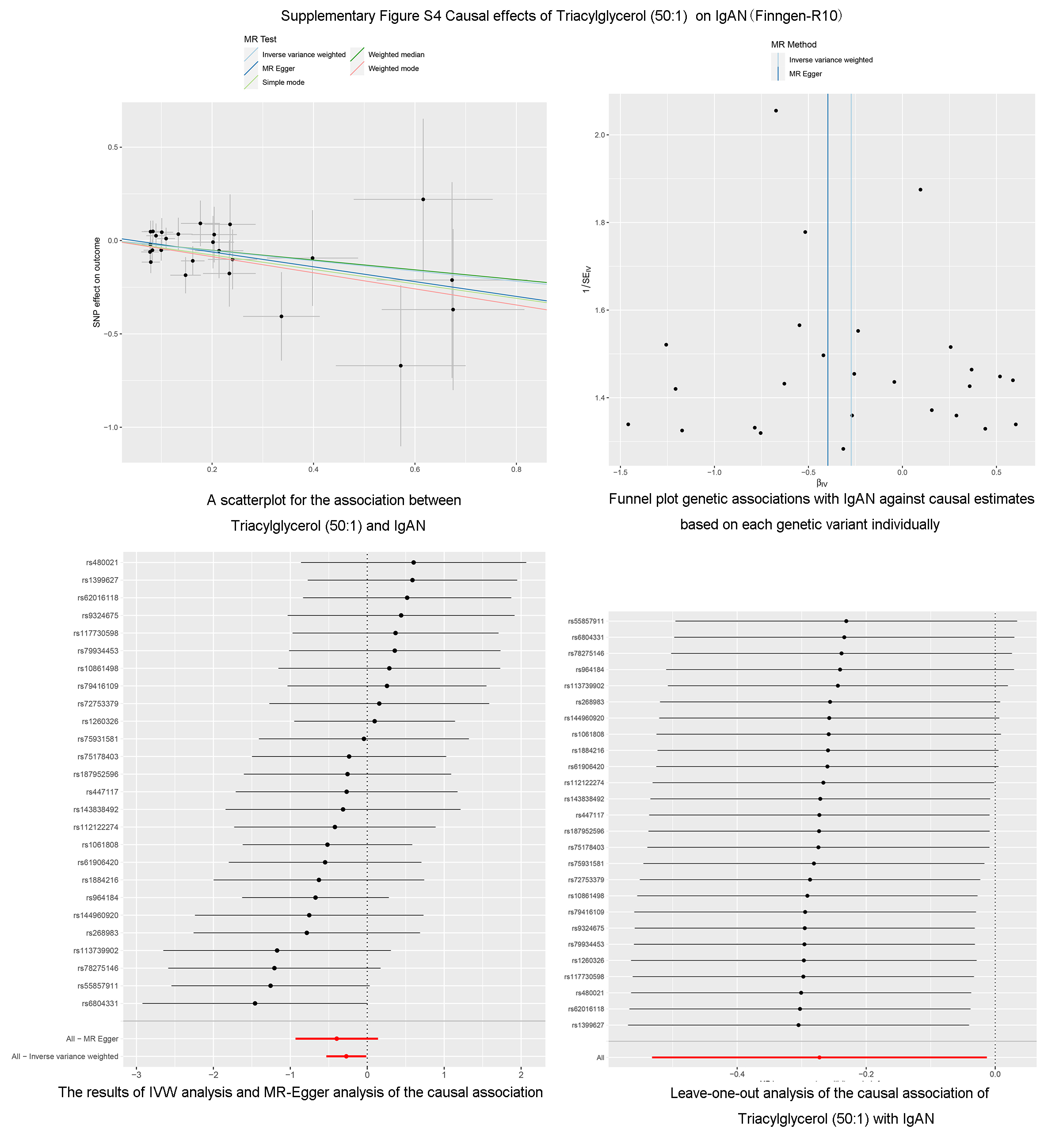

Supplement: Supplementary Figure S4.jpg [file IRNF_A_2498631_SM5573.jpg]

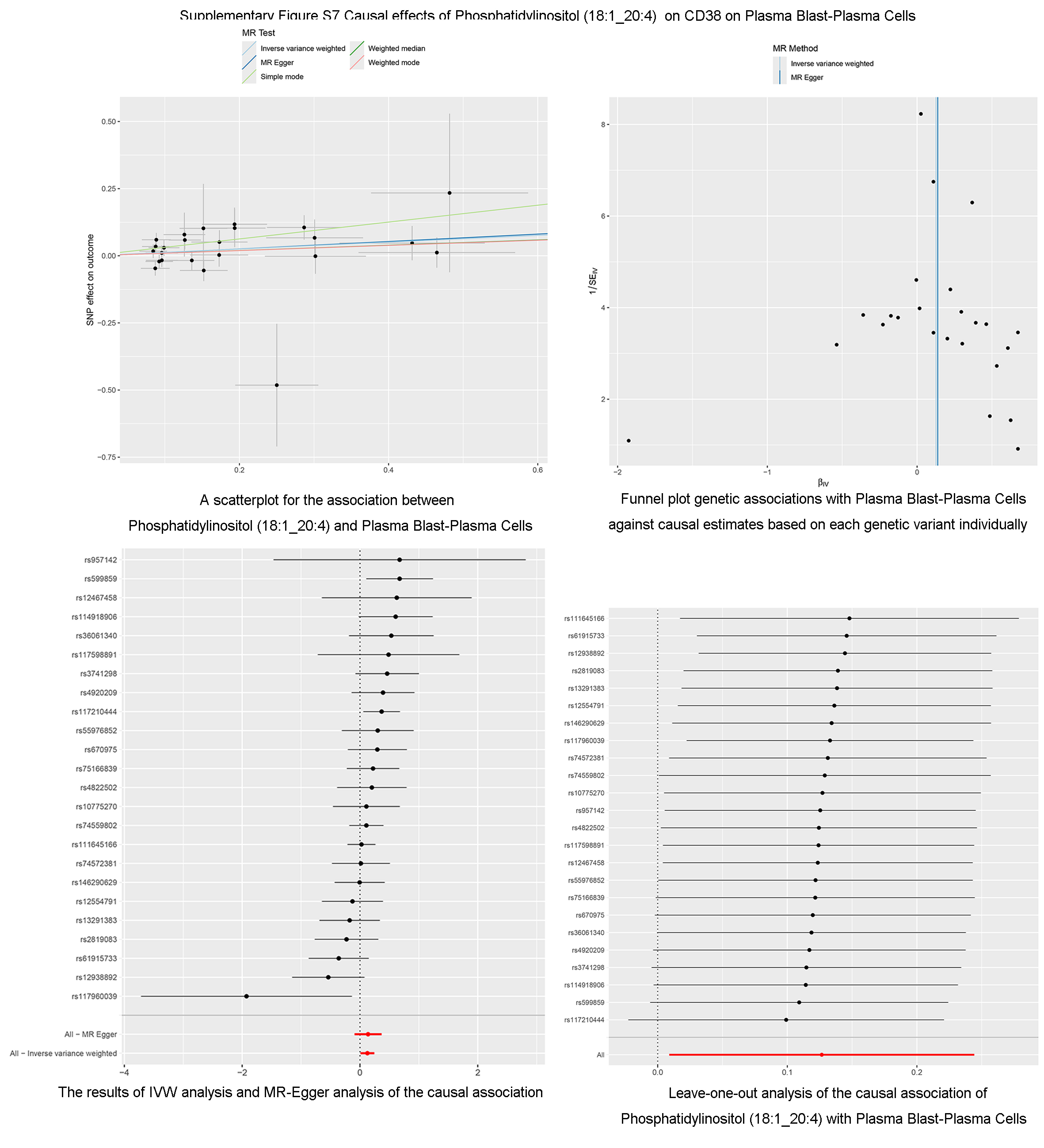

Supplement: Supplementary Figure S7.jpg [file IRNF_A_2498631_SM5571.jpg]

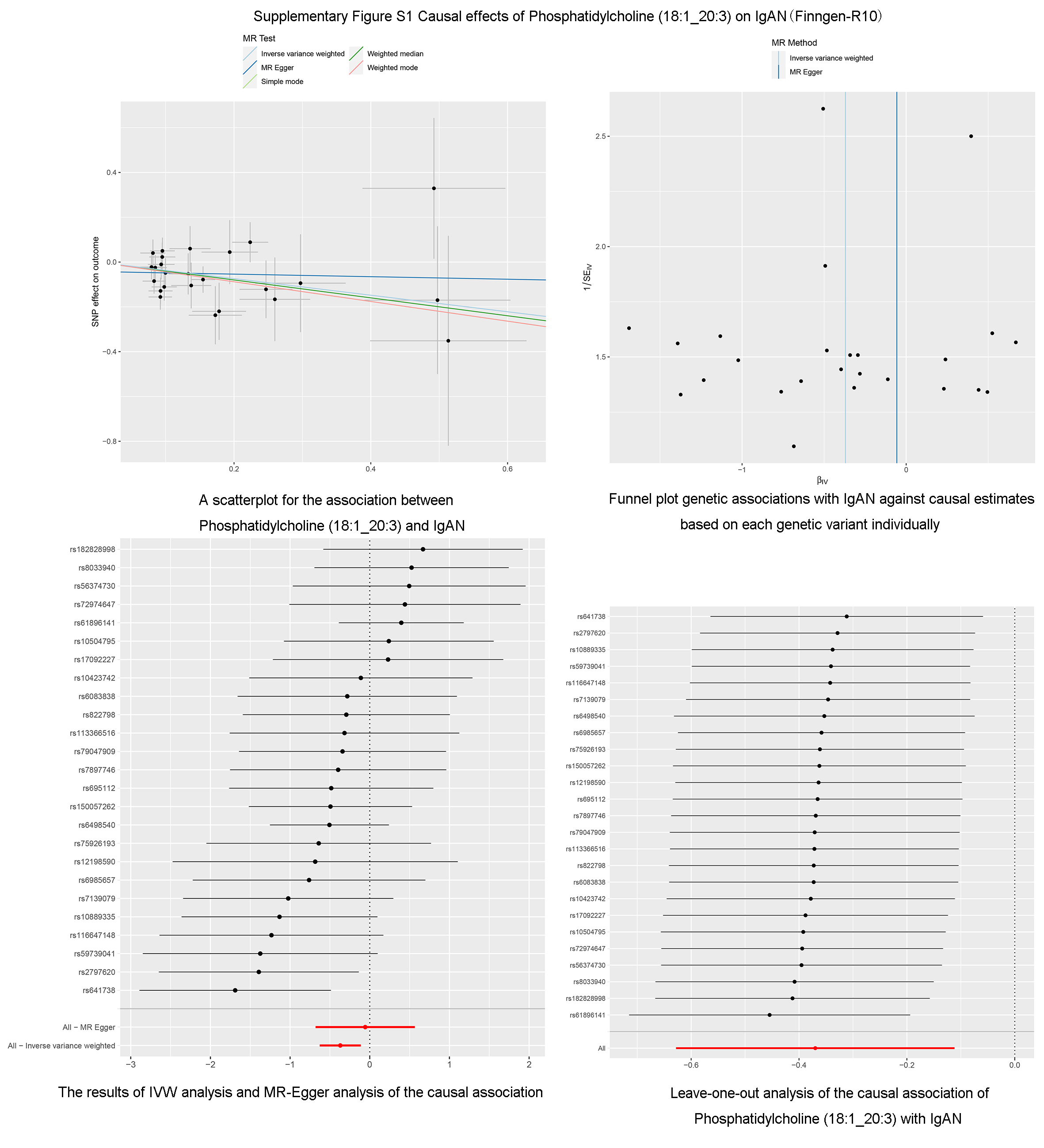

Supplement: Supplementary Figure S1.jpg [file IRNF_A_2498631_SM5570.jpg]

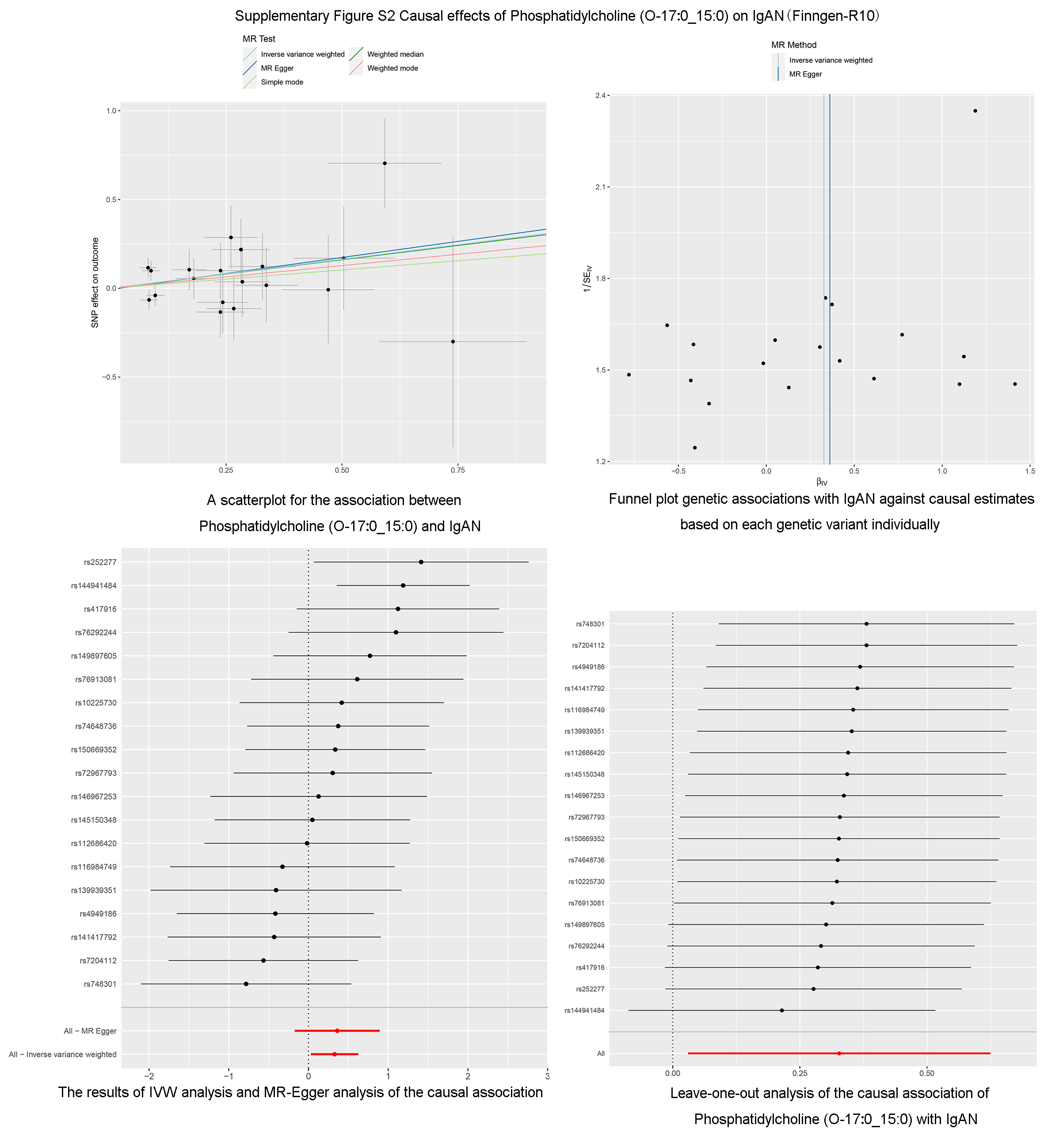

Supplement: Supplementary Figure S2.jpg [file IRNF_A_2498631_SM5569.jpg]

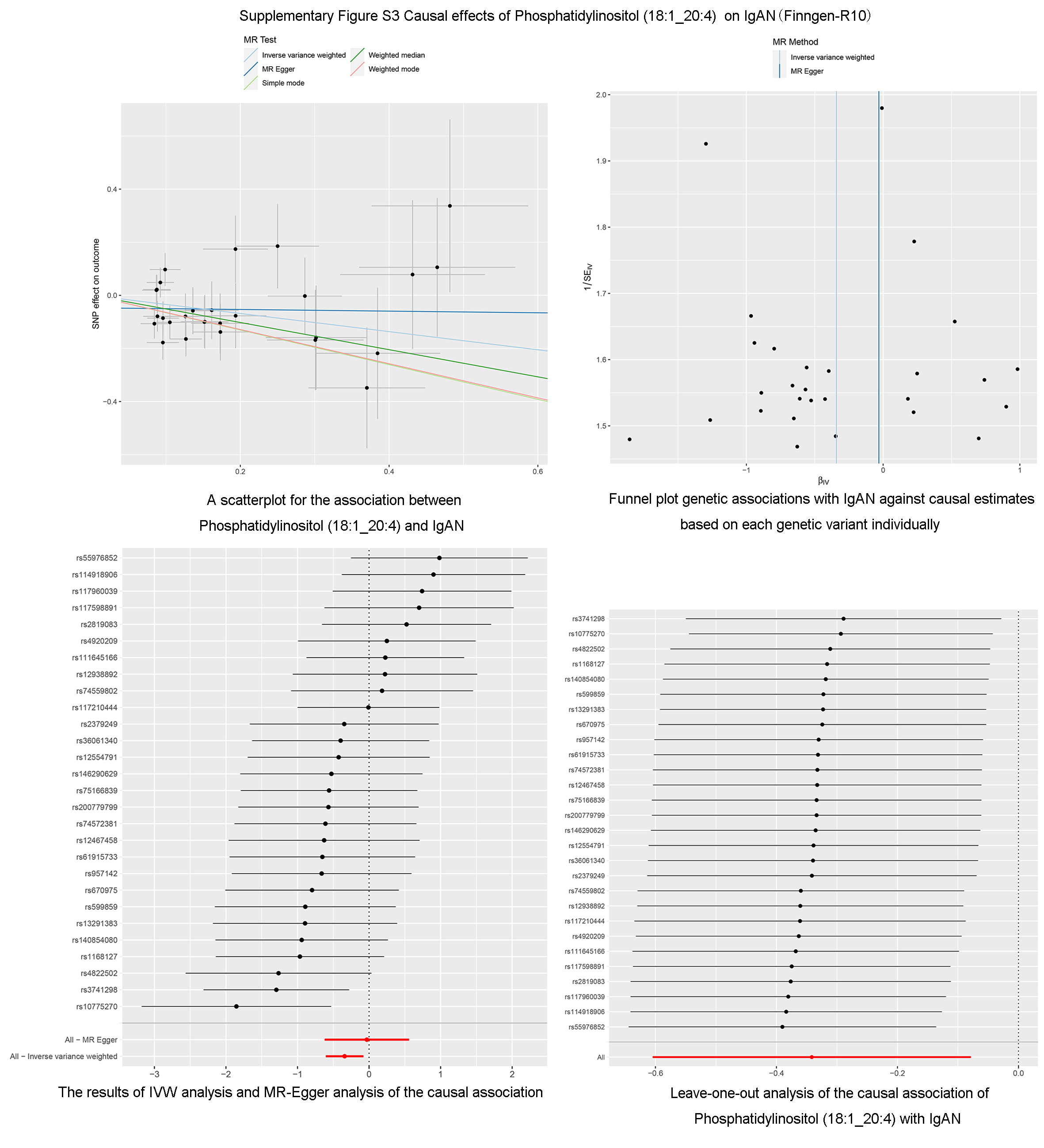

Supplement: Supplementary Figure S3.jpg [file IRNF_A_2498631_SM5568.jpg]

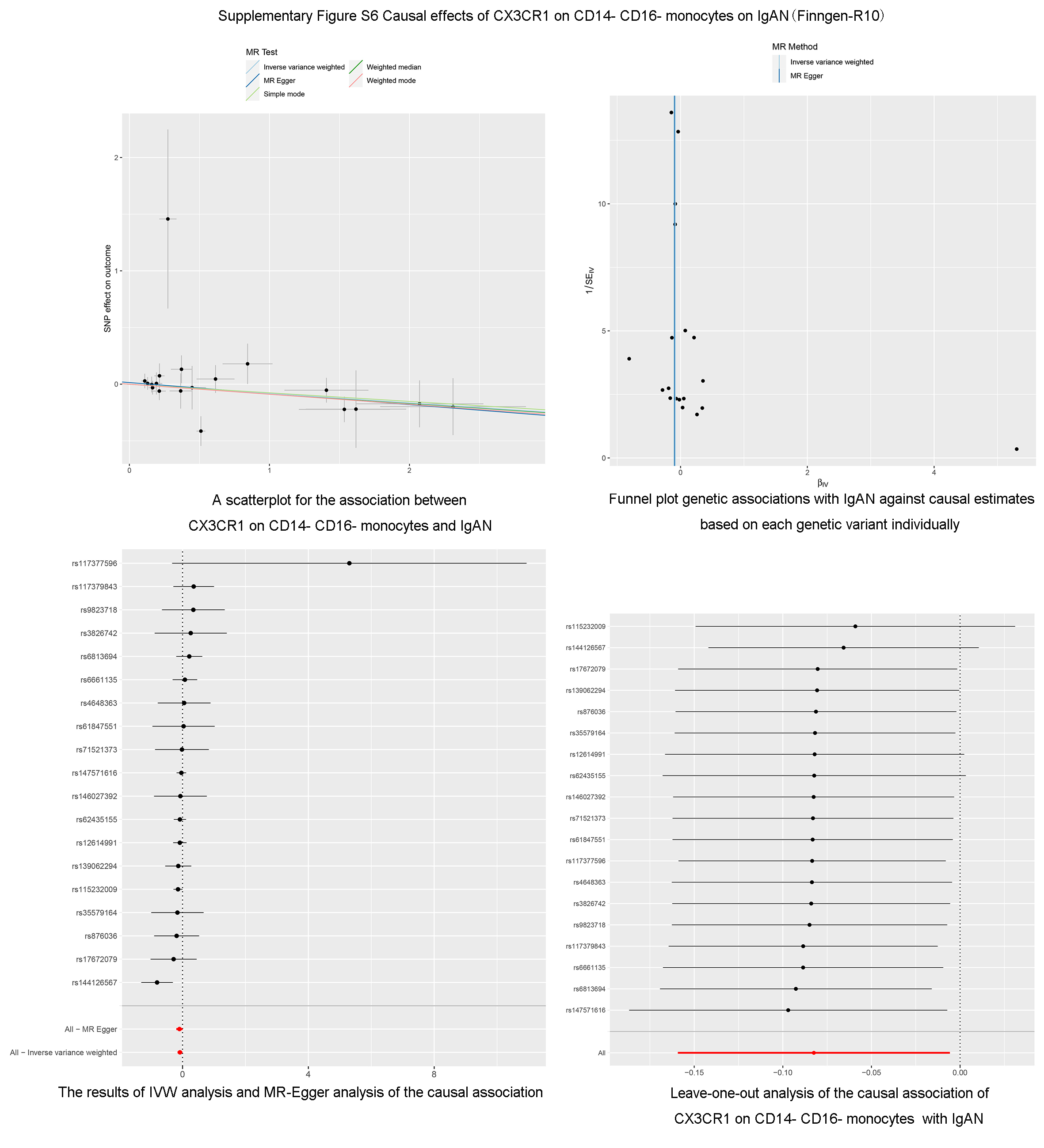

Supplement: Supplementary Figure S6.jpg [file IRNF_A_2498631_SM5567.jpg]
